# Dual-Tunable Memristor Based on Carbon Nanotubes and Graphene Quantum Dots

**DOI:** 10.3390/nano11082043

**Published:** 2021-08-11

**Authors:** Lu Wang, Jing Yang, Yukai Zhang, Dianzhong Wen

**Affiliations:** HLJ Province Key Laboratory of Senior-Education for Electronic Engineering, School of Electronic Engineering, Heilongjiang University, Harbin 150080, China; 2181219@s.hlju.edu.cn (J.Y.); 2191278@s.hlju.edu.cn (Y.Z.); wendianzhong@hlju.edu.cn (D.W.)

**Keywords:** multiwalled carbon nanotubes, graphene quantum dots, tunable memristor, UV illumination

## Abstract

Nanocarbon materials have the advantages of biocompatibility, thermal stability and chemical stability and have shown excellent electrical properties in electronic devices. In this study, Al/MWCNT:GQD/ITO memristors with rewritable nonvolatile properties were prepared based on composites consisting of multiwalled carbon nanotubes (MWCNTs) and graphene quantum dots (GQDs). The switching current ratio of such a device can be tuned in two ways. Due to the ultraviolet light sensitivity of GQDs, when the dielectric material is illuminated by ultraviolet light, the charge capture ability of the GQDs decreases with an increasing duration of illumination, and the switching current ratio of the device also decreases with an increasing illumination duration (10^3^–10). By exploiting the charge capture characteristics of GQDs, the trap capture level can be increased by increasing the content of GQDs in the dielectric layer. The switching current ratio of the device increases with increasing GQD content (10–10^3^). The device can be programmed and erased more than 100 times; the programmable switching state can withstand 10^5^ read pulses, and the retention time is more than 10^4^ s. This memristor has a simple structure, low power consumption, and enormous application potential for data storage, artificial intelligence, image processing, artificial neural networks, and other applications.

## 1. Introduction

Resistive random access memory is a kind of nonvolatile memory with excellent application prospects [1,2,3,4,5]. Because of its advantages, such as a simple structure, simple processing, low cost, low power consumption, and compatibility with CMOS processes, it has received wide attention [6,7,8,9,10,11,12]. At present, the materials used to fabricate memristors include perovskites [13], metal oxides [14], natural biomaterials [7,15,16], and organic dielectric materials [17]. Compared with other materials, carbon has the advantages of biocompatibility, higher thermal stability, and a stronger chemical stability. Many memristors prepared with carbon nanotubes as a dielectric layer have been reported in the literature [18,19,20]. Russo prepared novel carbon nanotubes (CNWs) on a fluorine-doped tin oxide (FTO) substrate. The electrical properties of corresponding memristors could be designed by controlling the parameters of the carbon nanotubes [21]. Thomas studied the electrical properties of a resistive switching device made of amorphous carbon, where the switching behavior was caused by a conductive pathway formed by transitions between the hybrid states of sp2 and sp3 [22]. Liu fabricated an ITO/SC-MCNT/PI/Al memristor based on carbon composite nanofibers (SC-MCNTs). The device had a switching current ratio of 10^3^–10^4^, a low threshold voltage and a resistance retention time of 3000 s [23].

Graphene quantum dots (GQDs) are a new zero-dimensional carbon-based material with excellent optical properties, good water solubility, low cost, good biocompatibility and many other advantages [24]. Their quantum well structures naturally form charge capture centers, which are highly beneficial for regulating the resistance of a device [25]. At present, a number of applications of graphene quantum dots in memristors have been reported in the literature [26,27]. Al/OCQD-GO/ITO memristors were prepared by using a carbon oxide quantum dot (OCQD-GO) nanocomposite as the dielectric layer of memristors. With an increasing OCQD concentration in the dielectric layer, the resistance value and threshold voltage of the high-resistance state increased [28]. It has been proven in the literature that graphene quantum dots are also UV sensitive. Lu studied the resistance switching behavior of composite photonic biofilms consisting of carbon quantum dots and silk fibroin. It was found that the carbon quantum dots, with their phototunable charge trapping ability, played an important role in the resistance switching characteristics of memristors and that the synergistic effect of photoelectric and photo-control effectively enhanced the internal electric field of such a device and reduced the threshold voltage [11]. Memristors prepared by Lin using graphene oxide and a nitrogen-doped carbon quantum dot (NCQD) nanocomposite material also showed resistance switching behavior. After ultraviolet light irradiation of such a device, multiple weak conductive wires had formed, and the device showed simulated resistance switching with continuous conductivity changes [29].

The surfaces of multiwalled carbon nanotubes host a large number of surface groups. As the number of wall layers of carbon nanotubes increases, their defects and chemical reactivity are enhanced, and it becomes easier for them to participate in electrical reactions in a device. Graphene quantum dots exhibit resistance regulation capabilities and UV sensitivity. In the present study, based on the excellent properties of multiwalled carbon nanotubes and graphene quantum dots, Al/MWCNT:GQD/ITO memristors were fabricated and their switching behavior was investigated. The effects of MWCNTs doped with different contents of GQDs on the switching current ratio and stability of the device are discussed. Then, the effects of different irradiation durations on the switching current ratio of the device after ultraviolet irradiation of the composite are studied.

## 2. Materials and Methods

### 2.1. Materials

For this experiment, 10–30 μm long multiwalled carbon nanotubes (purity > 95 wt%) and graphene quantum dots with a diameter of 15 nm and a concentration of 1 mg/mL (purity ~ 80%) were purchased from Tanfeng Tech. Inc., Suzhou, China, with a C content of 46.22%, an O content of 49.91%, and a H content of 3.87%.

### 2.2. Composite Material Preparation

Carbon nanotubes and deionized water were prepared in a 1 mg/mL dispersed solution, and ultrasonic vibration was carried out for 4 h. Then, graphene quantum dots were mixed into the carbon nanotube dispersion to prepare MWCNTs with mass ratios of 1:0.125, 1:0.25, and 1:0.5, and ultrasonic oscillations were carried out for 1 h. To study the influence of ultraviolet light on the resulting device, the mixed solution of carbon nanotubes and graphene quantum dots with a mass ratio of 1:0.5 was irradiated for 10 min, 20 min, and 30 min under an ultraviolet lamp with a wavelength of 365 nm.

### 2.3. Fabrication of the Memory Devices

ITO glass was successively placed in acetone, ethanol and deionized water for ultrasonic cleaning for 20 min each to remove surface contaminants. All mixed solutions were spin coated at 2000 rpm for 20 s and dried at 80 °C for 40 min. The top electrode (Al) was then deposited via the vacuum evaporation method.

### 2.4. Characterization

The microstructure of the MWCNTs was observed using a JEM-2100 transmission electron microscope (Joel, Tokyo, Japan). The Raman spectra of the MWCNTs and GQDs were studied using a DXR2XI Raman system (Thermo Fisher Scientific, Waltham, MA, USA) at a 532 nm excitation wavelength. The devices were electrically tested using a Keithley 4200-SCS Semiconductor Parameter Tester (Tektronix, Solon, OH, USA).

## 3. Results

**Figure 1:**
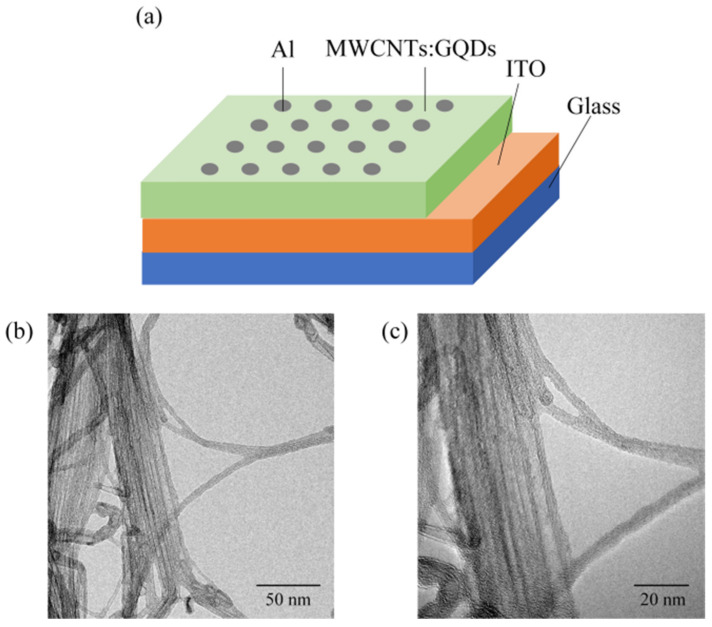
(**a**) Al/MWCNT:GQD/ITO device structure; TEM images of MWCNTs at (**b**) low resolution and (**c**) high resolution.

A diagram of the Al/MWCNT:GQD/ITO structure is shown in Figure 1a. The microstructure of the carbon nanotubes was observed at magnifications of 10,000 and 20,000 times via transmission electron microscopy, as shown in Figure 1b,c. It can be seen that the multiwalled carbon nanotubes have hollow tubular structures.

**Figure 2:**
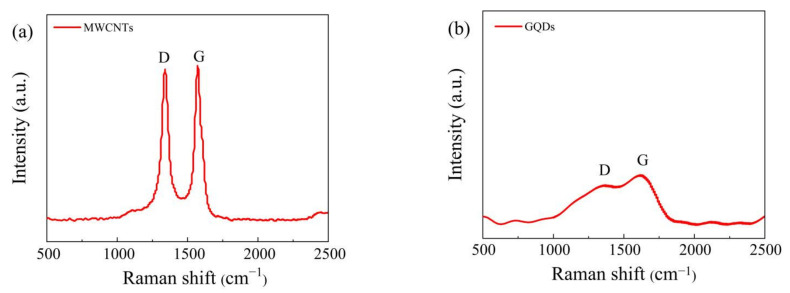
Raman spectra of (**a**) MWCNTs and (**b**) GQDs.

The Raman spectra of the MWCNTs and GQDs are shown in Figure 2. As shown in Figure 2a, the spectrum of the MWCNTs exhibits two characteristic peaks, namely, the D-band peak (1340 cm^−1^) generated by defects and the characteristic G-band peak (1570 cm^−1^) generated by the in-plane vibration of sp2 carbon atoms. The intensity ratio of the D-band peak to the G-band peak of the CNTs (ID/IG) is 0.98, indicating the existence of structural defects and disorder in the MWCNTs [18,22]. As shown in Figure 2b, the spectrum of the GQDs exhibits a very weak D-band peak at 1370 cm^−1^, indicating the existence of local defects and lattice disorder, and another characteristic G-band peak is located near 1620 cm^−1^. The intensity ratio (ID/IG) of the D peak to the G peak is 0.80, indicating high purity and a low oxidation degree of the GQDs [30,31].

**Figure 3:**
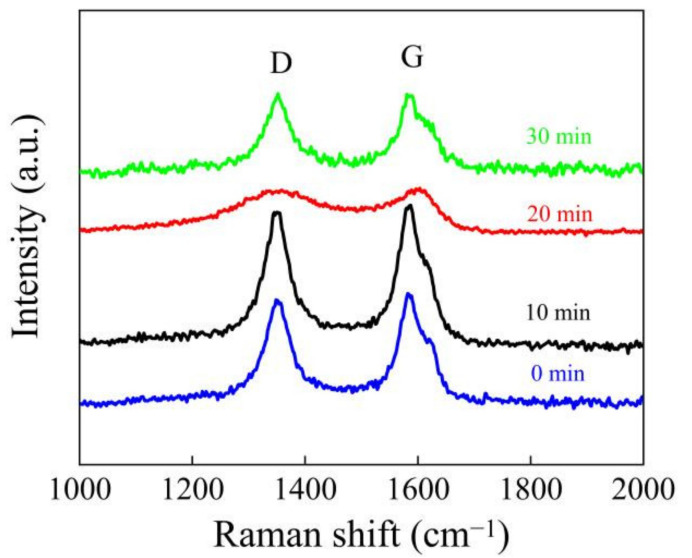
Raman spectra of mixed solutions after different UV irradiation times.

To study the influence of UV light irradiation on the dielectric layer, we irradiated a mixed solution of MWCNTs and GQDs with a mass ratio of 1:0.5 at 365 nm for 0, 10, 20, and 30 min and then tested the Raman spectra of the mixed solution, as shown in Figure 3. The D-band peak is located at 1353 cm^−1^, and the G-band peak is located at 1582 cm^−1^. The ratio of the D-band intensity to the G-band intensity (ID/IG) increased significantly under irradiation, from 0.91 to 1.01. It has been proven that the increase in sp2 carbon atoms and the decrease in oxygen-containing groups that occur in the process of UV irradiation lead to a decrease in the carrier trap concentration and a decrease in the high resistance value of a device [11,29].

**Figure 4:**
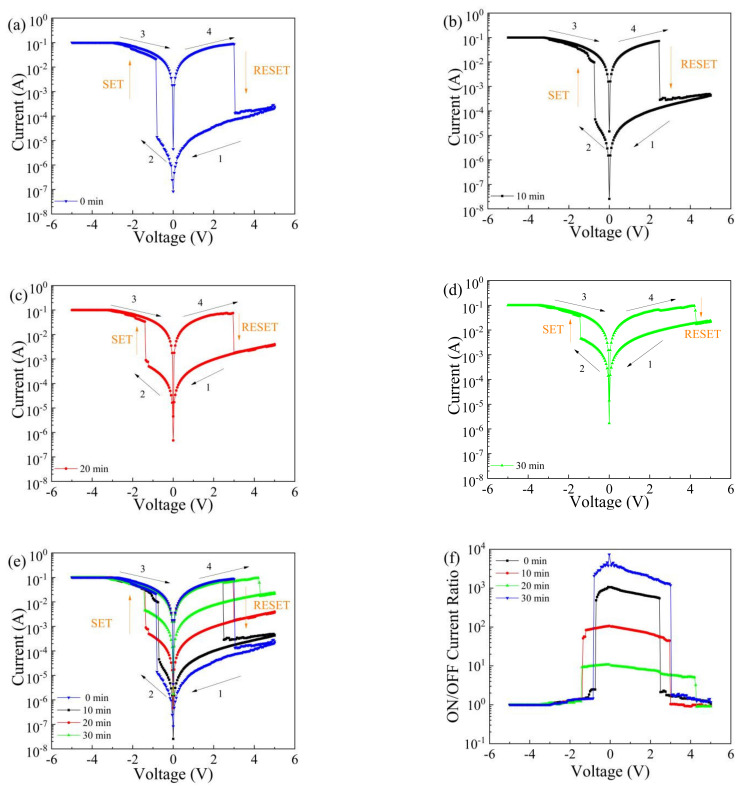
I–V characteristic curves of an Al/MWCNT:GQD/ITO device (MWCNT:GQD mass ratio = 1:0.5) under different durations of UV light irradiation: (**a**) 0 min, (**b**) 10 min, (**c**) 20 min, and (**d**) 30 min; (**e**) superposition of I–V characteristic curves after different UV irradiation durations; (**f**) ON/OFF current ratios after different UV irradiation durations.

To study the effect of the UV irradiation time on the electrical properties of Al/MWCNT:GQD/ITO devices, we tested the devices at room temperature using a semiconductor parameter tester. During the test, the ITO electrode was grounded, a scanning voltage of approximately −5 V to +5 V was applied to the Al electrode, and the limiting current was set to 0.1 A to prevent damage to the device. The voltage scanning direction is shown in the figure. Figure 4a–d show the I–V characteristic curves of the Al/MWCNT:GQD/ITO device with a mass ratio of 1:0.5 between the MWCNTs and GQDs after 0, 10, 20 and 30 min of UV irradiation. The devices show bipolar resistance switching characteristics. The superposition of the I–V curves after different UV irradiation times is shown in Figure 4e, from which it can be seen that the initial resistance of the device decreases from 2.46 × 10^4^ Ω to 3.00 × 10^2^ Ω with increasing irradiation duration. The relationship between the ON/OFF current ratio of the device and the UV irradiation time is shown in Figure 4f. The maximum switching current ratios of the device after illumination times of 0 min, 10 min, 20 min, and 30 min are 4.58 × 10^3^, 1.06 × 10^3^, 1.18 × 10^2^, and 10.77, respectively. The switching current ratio decreases with increasing UV illumination time because some of the traps in the dielectric layer are filled after illumination, which leads to a reduction in the trap capture depth and thus a reduction in the switching current ratio.

**Figure 5:**
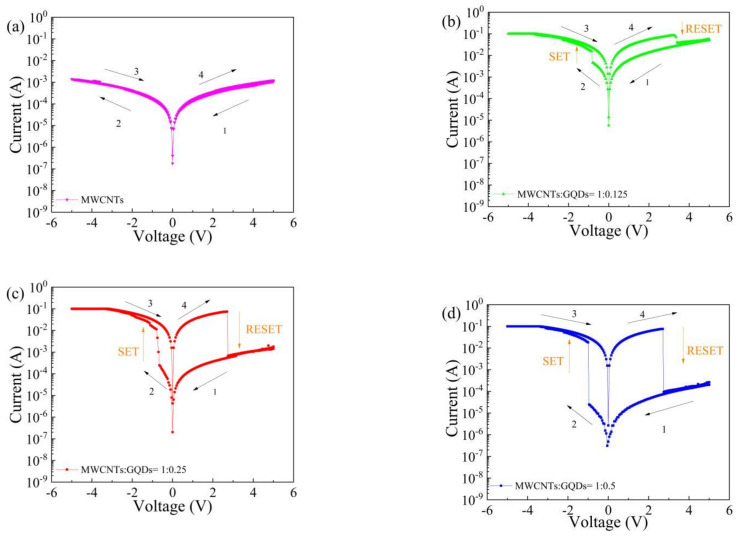

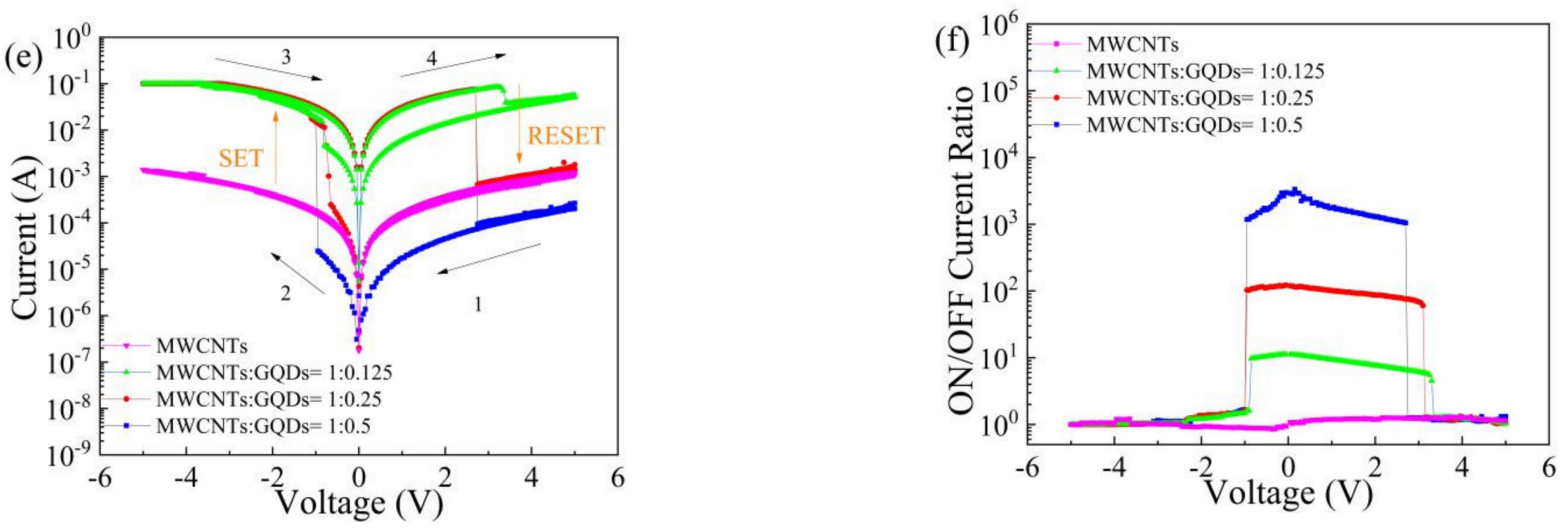
Electrical characteristics of Al/MWCNT:GQD/ITO devices with different mass ratios between the MWCNTs and GQDs: (**a**) MWCNTs only, (**b**) MWCNT:GQD mass ratio = 1:0.125, (**c**) MWCNT:GQD mass ratio = 1:0.25, (**d**) MWCNT:GQD mass ratio = 1:0.5; (**e**) superposition of I–V characteristic curves of devices with different dielectric layer concentrations; (**f**) ON/OFF current ratio.

The influence of different contents of GQDs in the dielectric layer on the electrical characteristics of the device was tested using a semiconductor parameter tester, as shown in Figure 5a–d. During the test, the ITO electrode was grounded, a scanning voltage of approximately −5 V to 5 V was applied to the Al electrode, and the compliance current was set to 0.1 A to prevent damage to the equipment. The test results for the Al/MWCNT:GQD/ITO device with a MWCNT:GQD mass ratio of 1:0.5 are shown in Figure 5d. Initially, the device remained in the HRS. When the voltage reached −0.95 V, the current increased from 2.47 × 10^−5^ A to 1.82 × 10^−2^ A, and the device switched from the HRS to the LRS, corresponding to the process of data writing. When the voltage reached 2.70 V, the current decreased from 7.58 × 10^−2^ A to 9.33 × 10^−5^ A, and the device recovered from the LRS to the HRS, corresponding to the process of data erasure. The same voltage scanning method was also used to test the other devices with different dielectric layer concentrations, and the results show that the Al/MWCNT/ITO device does not have resistance switching characteristics, while the GQD-doped memristors all show bipolar resistance switching characteristics. The ON/OFF current ratios of the devices with different dielectric layer concentrations are shown in Figure 5f. With an increase in the content of GQDs, the initial resistance of the device increases. The ON/OFF current ratio of the Al/MWCNT/ITO device is 1, and the maximum ON/OFF current ratios of the Al/MWCNT:GQD/ITO devices with MWCNT:GQD mass ratios of 1:0.125, 1:0.25, and 1:0.5 are 11.12, 1.22 × 10^2^, and 3.34 × 10^3^, respectively, indicating that the ON/OFF current ratio of the device becomes increasingly larger with increasing GQD content in the dielectric layer. Thus, the ON/OFF current ratio of such a device can be tuned by adjusting the content of graphene quantum dots to achieve multilevel data storage.

**Figure 6:**
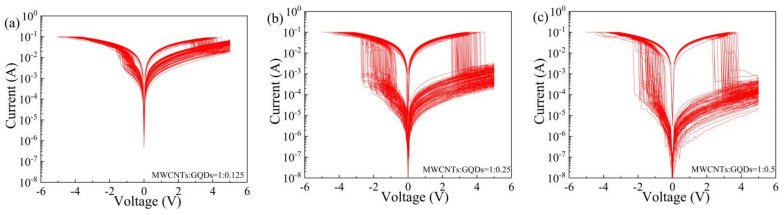
I–V characteristic curves of Al/MWCNT:GQD/ITO devices over 100 continuous voltage scans: (**a**) MWCNT:GQD mass ratio = 1:0.125, (**b**) MWCNT:GQD mass ratio = 1:0.25, and (**c**) MWCNT:GQD mass ratio = 1:0.5.

**Figure 7:**
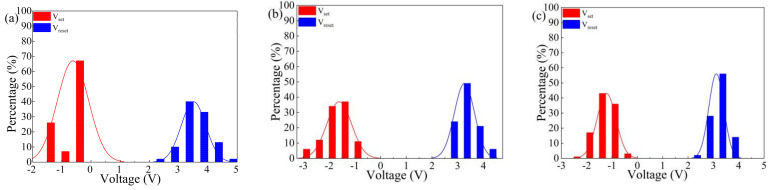
Vset and Vreset distributions of devices with different mass ratios between the MWCNTs and GQDs: (**a**) MWCNT:GQD mass ratio = 1:0.125, (**b**) MWCNT:GQD mass ratio = 1:0.25, and (**c**) MWCNT:GQD mass ratio = 1:0.5.

To explore the continuous erasability characteristics of Al/MWCNT:GQD/ITO devices with three different dielectric layer concentrations, we performed continuous voltage scanning on the same memory unit on each device, with the results shown in Figure 6. A single memory unit of each of these three kinds of memristors can maintain its switching characteristics for more than 100 cycles under continuous voltage scanning. The threshold voltage is an important parameter of a memristor [3,32]. The distributions of Vset and Vreset for the Al/MWCNT:GQD/ITO devices under 100 consecutive voltage scans are shown in Figure 7. The average Vset and Vreset values of the Al/MWCNT:GQD/ITO device with a MWCNT:GQD mass ratio of 1:0.125 are −0.62 V and 3.47 V, respectively, with corresponding standard deviations of 0.53 and 0.41; the average Vset and Vreset values of the Al/MWCNT:GQD/ITO device with a MWCNT:GQD mass ratio of 1:0.25 are −1.60 V and 3.28 V, respectively, with corresponding standard deviations of 0.48 and 0.39; and the average Vset and Vreset values of the Al/MWCNT:GQD/ITO device with a MWCNT:GQD mass ratio of 1:0.5 are −1.23 V and 3.12 V, respectively, with corresponding standard deviations of 0.37 and 0.29. These results show that the threshold voltage distributions of the Al/MWCNT:GQD/ITO device with a MWCNT:GQD mass ratio of 1:0.5 are the most stable.

**Figure 8:**
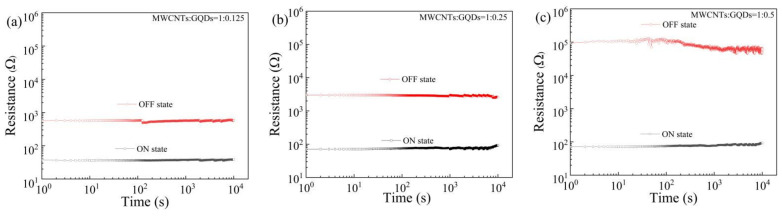
Retention times of Al/MWCNT:GQD/ITO devices with different mass ratios between the MWCNTs and GQDs: (**a**) MWCNT:GQD mass ratio = 1:0.125, (**b**) MWCNT:GQD mass ratio = 1:0.25, and (**c**) MWCNT:GQD mass ratio = 1:0.5.

**Figure 9:**
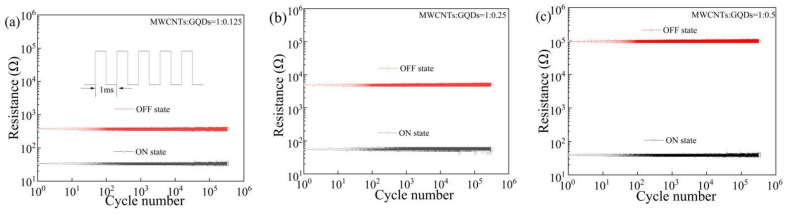
Cycle endurance of Al/MWCNT:GQD/ITO devices with different mass ratios between the MWCNTs and GQDs: (**a**) MWCNT:GQD mass ratio = 1:0.125, (**b**) MWCNT:GQD mass ratio = 1:0.25, and (**c**) MWCNT:GQD mass ratio = 1:0.5.

The data retention time is a key performance metric of resistive switching storage devices. The Al/MWCNT:GQD/ITO devices with different dielectric layer concentrations were tested under a constant voltage of 1 V to measure the resistance retention time over 10^4^ s, as shown in Figure 8a–c, and the results indicate that the devices show good data storage performance. The endurance performance in various resistance states observed for the Al/MWCNT:GQD/ITO devices is presented in Figure 9a–c. Under continuous pulse cycles (with a pulse period and pulse width of 1 ms and 0.5 ms, respectively), the HRS and LRS remained stable for more than 10^5^ pulses, indicating excellent device stability.

**Figure 10:**
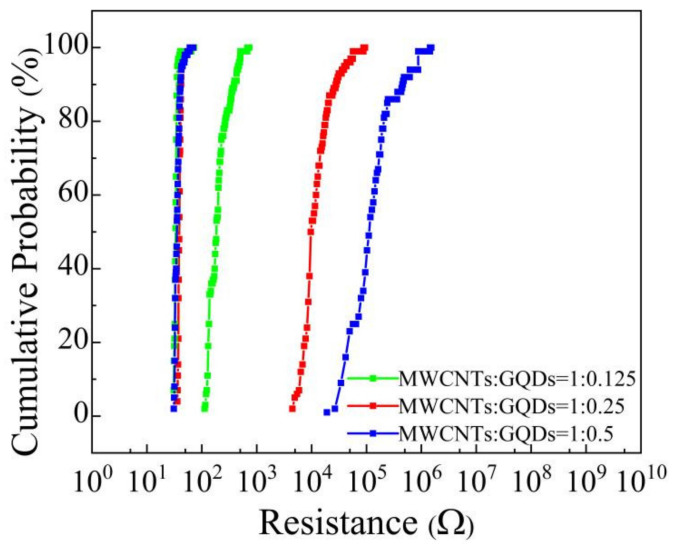
Cumulative probability distributions of resistance for Al/MWCNT:GQD/ITO devices.

To investigate the uniformity of the resistance of Al/MWCNT:GQD/ITO devices, we studied the cumulative probability distributions of the resistance for the devices with MWCNT:GQD mass ratios of 1:0.125, 1:0.5 and 1:0.5 under 100 continuous cycles at 1 V, as shown in Figure 10. The results show that the resistance uniformity of these devices is excellent.

**Figure 11:**
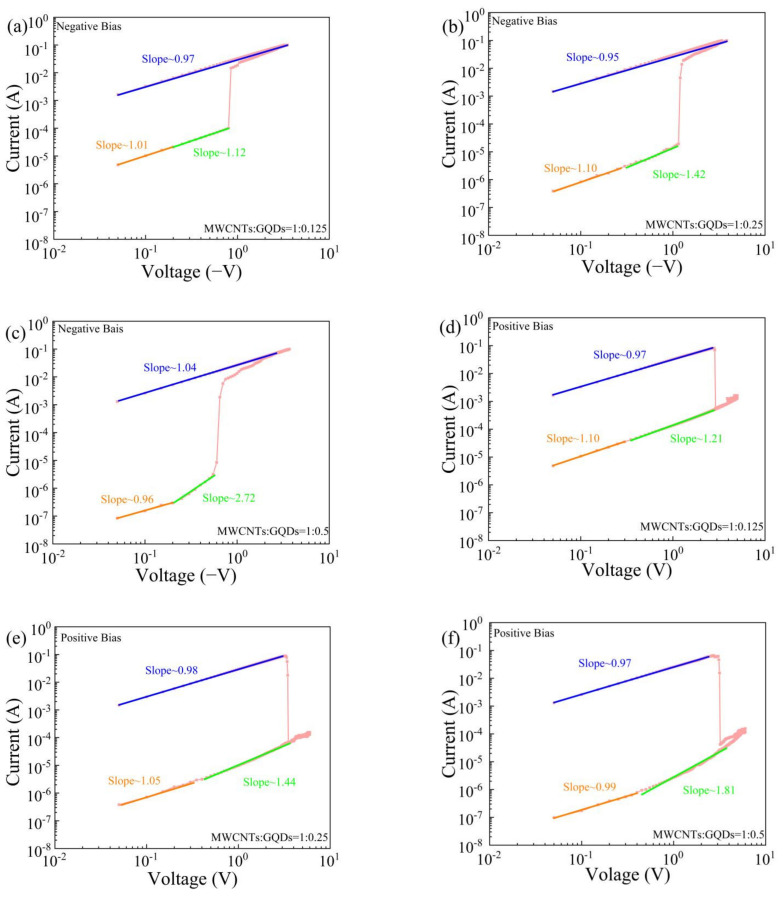
Log(I)–log(V) plots with fitted curves for Al/MWCNT:GQD/ITO devices: (**a**–**c**) negative voltage and (**d**–**f**) positive voltage.

To understand the current transfer mechanism, the I–V characteristic curves were drawn in log-log coordinates. The I–V curves of the Al/MWCNT:GQD/ITO memristors in the negative voltage region are shown in Figure 11a–c. In particular, the I–V curve of the memristor with a MWCNT:GQD mass ratio of 1:0.5 is shown in Figure 11c. It can be seen that when the device is in the LRS, the I–V curve is linear in both the low-voltage region and the high-voltage region, i.e., I ∝ V, which indicates that the current transmission follows ohmic conduction [10,33]. When the device is in the HRS, the fitted I–V curve can be divided into two parts. In the region corresponding to a voltage of less than 0.20 V, the slope of the I–V curve is approximately equal to 1, and the I–V curve is nearly linear. Outside this region, where the voltage is greater than 0.20 V, the slope of the I–V curve is approximately equal to 2, which is consistent with the space-charge-limited current (SCLC) conduction model related to trap filling [34]. When the concentration of graphene quantum dots in the device is low, the slope of the I–V curve is less than 2, consistent with a weak SCLC conduction mechanism, which may be related to a shallow capture level in the dielectric layer, indicating that GQDs serving as charge capture centers may act as deeper traps [28]. The fitted I–V curves under a positive voltage are shown in double-logarithmic coordinates in Figure 11d–f. The devices exhibit ohmic conduction at low resistance, while the SCLC transmission mechanism dominates at high resistance, consistent with the current transmission mechanism in the negative voltage region.

**Figure 12:**
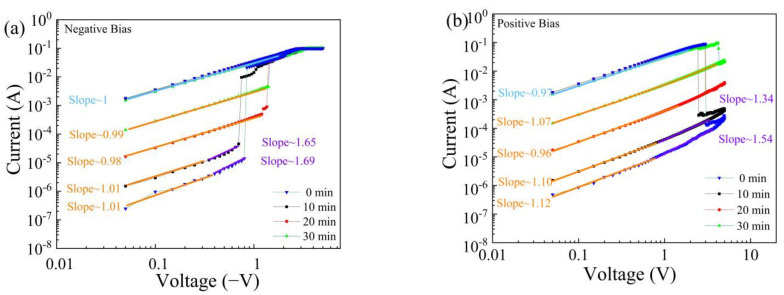
Log(I)–log(V) plots with fitted curves for Al/MWCNT:GQD/ITO devices after different UV irradiation times: (**a**) negative voltage and (**b**) positive voltage.

To study the effects of ultraviolet light on the current transmission mechanism of the devices, the I–V characteristic curves of the devices after different irradiation times were also plotted in a double-logarithmic coordinate system. The results of applying a negative voltage to the memristors after ultraviolet illumination are shown in Figure 12a. The I–V characteristic curve is linear in the LRS, indicating that the conduction current transmission follows Ohm’s law. In the HRS, the slope of the current-voltage curve in the low-voltage region is approximately equal to 1, which is close to a linear relationship. In the high-voltage region, the slope of the I–V curve is approximately equal to 2, which is consistent with SCLC transmission mechanism. With increasing illumination time, the traps in the GQDs are partially filled [11,29], the trap depth of the device decreases, the SCLC transmission mechanism weakens, and the slope of the I–V curve decreases. The results when a positive voltage is applied are shown in Figure 12b. At low resistance, the devices exhibit ohmic conduction, while at high resistance, the devices are consistent with SCLC transmission mechanism.

**Figure 13:**
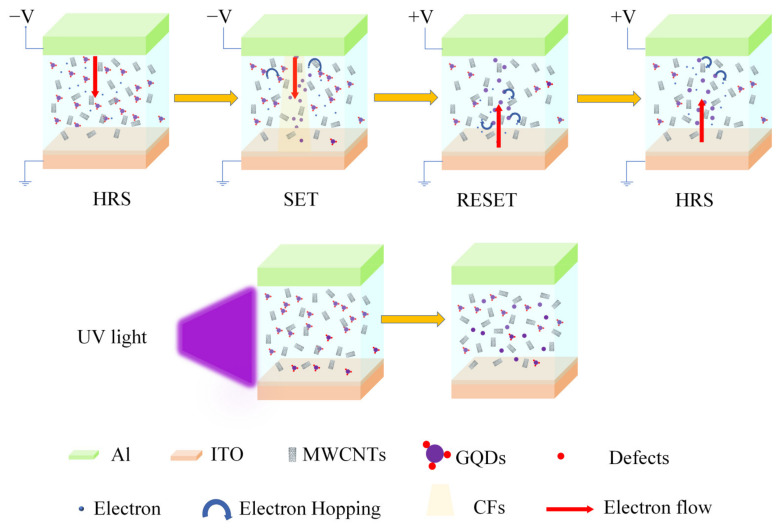
Conduction mechanism of an Al/MWCNT:GQD/ITO memristor and the effect of UV on the dielectric film.

The conduction mechanism of an Al/MWCNT:GQD/ITO device is shown in Figure 13. Due to the charge-trapping effect of the GQDs, more oxygen-containing groups will be introduced into the CNTs to increase the energy band barrier, thus deepening the electron trapping depth of the composite film and increasing the initial resistance of the device. When a negative voltage is applied to the upper electrode (Al), electrons are injected into the composite film from the upper electrode and captured by traps. The top electrode provides an electron source to continuously inject electrons, and electrons can reach the bottom electrode through randomly distributed trap centers [35,36]. At this time, a conductive path is formed inside the device, and the current sharply increases. When a positive voltage is applied to the upper electrode (Al), the trapped electrons in the composite film move towards the top electrode under the action of the electric field, the conductive path breaks, and the current sharply decreases [37]. With increasing GQD content in the dielectric layer, more defects are introduced, resulting in an increase in the trap depth, the initial resistance and the switching current ratio. Under UV irradiation, the traps in the composite film become partially filled in the initial state; hence, the number of defects decreases with increasing illumination time [11], the initial resistance of the device decreases, and the switching current ratio decreases.

## 4. Conclusions

In summary, Al/MWCNT:GQD/ITO memristors with adjustable ON/OFF current ratios were prepared based on composites consisting of MWCNTs and GQDs. When a mixed solution of MWCNTs and GQDs is irradiated with UV light, the traps in the dielectric layer are partially filled. With increasing illumination time (0 min–30 min), the trap capture level in the dielectric layer decreases, the initial resistance of the device decreases, and the switching current ratio decreases. With increasing GQD content in the dielectric layer, more trap centers are introduced, the trap capture depth is increased, the initial resistance of the device increases, and the switching current ratio increases. The switching current ratios of Al/MWCNT:GQD/ITO devices with MWCNT:GQD mass ratios of 1:0.125, 1:0.25, and 1:0.5 were found to be 11.1, 1.2 × 10^2^, and 3.3 × 10^3^, respectively. A single memory cell of each memristor showed stable switching behavior over 100 cycles. The programmable on-off resistance state can withstand 10^5^ read pulses, and the retention time is more than 10^4^ s. These findings lay a foundation for faster speeds and lower power consumption in adjustable logic memory applications.

## Data Availability

The datasets used and/or analyzed during the current study are available from the corresponding author on reasonable request.
